# Systematic review of quantitative preference studies of treatments for rheumatoid arthritis among patients and at-risk populations

**DOI:** 10.1186/s13075-021-02707-4

**Published:** 2022-02-22

**Authors:** Gwenda Simons, Joshua Caplan, Rachael L. DiSantostefano, Jorien Veldwijk, Matthias Englbrecht, Karin Schölin Bywall, Ulrik Kihlbom, Karim Raza, Marie Falahee

**Affiliations:** 1 Present Address: Rheumatology Research Group, Institute of Inflammation and Ageing, University of Birmingham Research Laboratories, Queen Elizabeth Hospital, University of Birmingham, Birmingham, B15 2WB UK; 2 Janssen Pharmaceuticals, Titusville, NJ USA; 3 School of Health Policy & Management, Erasmus University Rotterdam, Rotterdam, The Netherlands; 4 Erasmus Choice Modelling Centre, Erasmus University Rotterdam, Rotterdam, The Netherlands; 5 Julius Center for Health and Primary Care, University Medical Center Utrecht, Utrecht, The Netherlands; 6 Freelance Data Scientist, Eckental, Germany; 7 Centre for Research Ethics and Bioethics, Department of Public Health and Caring Sciences, Uppsala University, Uppsala, Sweden; 8 Research into Inflammatory Arthritis Centre Versus Arthritis and MRC-Versus Arthritis Centre for Musculoskeletal Ageing Research, University of Birmingham, Birmingham, UK; 9 Sandwell and West Birmingham NHS Trust, Birmingham, UK

**Keywords:** Rheumatoid arthritis, Treatment preferences, Preventive treatment, Attributes, Systematic review

## Abstract

Treatments used for rheumatoid arthritis (RA) are under investigation for their efficacy to prevent RA in at risk groups. It is therefore important to understand treatment preferences of those at risk. We systematically reviewed quantitative preference studies of drugs to treat, or prevent RA, to inform the design of further studies and trials of RA prevention. Stated preference studies for RA treatment or prevention were identified through a search of five databases. Study characteristics and results were extracted, and the relative importance of different types of treatment attributes was compared across populations. Twenty three studies were included 20 of RA treatments (18 of patients; 2 of the general public) and 3 prevention studies with first-degree relatives (FDRs). Benefits, risks, administration method and cost (when included) were important determinants of treatment choice. A benefit was more important than a risk attribute in half of the studies of RA treatment that included a benefit attribute and 2/3 studies of RA prevention. There was variability in the relative importance of attributes across the few prevention studies. In studies with non-patient participants, attributes describing confidence in treatment effectiveness/safety were more important determinants of choice than in studies with patients. Most preference studies relating to RA are of treatments for established RA. Few studies examine preferences for treatments to prevent RA. Given intense research focus on RA prevention, additional preference studies in this context are needed. Variation in treatment preferences across different populations is not well understood and direct comparisons are needed.

**Supplementary Information:**

The online version contains supplementary material available at 10.1186/s13075-021-02707-4.

## Background

Rheumatoid arthritis (RA) is a common chronic inflammatory disease-causing joint pain, swelling, stiffness and fatigue. Persistent inflammation leads to erosive joint damage, functional impairment and disability [1–3]. RA is associated with significant extra-articular manifestations including cardiovascular disease [4] and therefore reduced life expectancy.

RA usually requires long-term treatment with associated risks of drug toxicity [5]. As a result, decisions to initiate or step up therapy are preference sensitive and understanding patient preferences is important to facilitate patient-centred strategies. Quantitative studies of patient preferences for RA treatments have been systematically reviewed [6].

There is increased research interest in the identification and treatment of ‘at risk’ individuals in order to delay or even prevent the onset of RA. The European League Against Rheumatism (EULAR) has identified terminology to describe five ‘at risk’ groups where preventive intervention may be possible. These are individuals without a diagnosis of RA who have either (i) genetic risk factors for RA, (ii) environmental risk factors for RA, (iii) systemic autoimmunity associated with RA, (iv) symptoms without clinical arthritis or (v) unclassified arthritis [7]. Core risk factors for RA have now been defined [8], and EULAR guidelines for conducting clinical trials and observational studies in individuals at risk of rheumatoid arthritis have been published [9].

Several completed and ongoing prevention trials are assessing the effectiveness of drugs currently used to treat RA, to delay or prevent the onset of RA (e.g. [10–14]). Initiatives to develop novel cellular treatments to prevent the RA are ongoing [15]. The decision to initiate a treatment to reduce the risk of developing RA is complex, as there is considerable uncertainty around the potential for benefit. Over recent years, some studies have begun to focus on the preferences of individuals at risk of RA for preventive treatments [16]. Understanding treatment preferences of those at risk is important to inform the development of ethical and efficient prevention trials and clinical translation. It is likely that treatment preferences of those who have a diagnosis of RA differ from those at risk.

Given the increasing focus on prevention of RA [8, 9], the present systematic review of studies to elicit preferences for RA treatments updates and extends the previous review [6] by including studies of the preferences of individuals who do not have RA, and those of ‘at risk’ populations for RA prevention [17]. This inquiry will explore differences between preference studies for RA treatment and prevention, and across different populations (patients, general public, at risk groups). Differences in study design and results will be examined. These findings will be informative for the design of efficient trials and healthcare strategies, and for attribute selection in further quantitative preference studies relating to RA prevention.

## Methods

This systematic review was conducted and reported in line with the Preferred Reporting Items for Systematic Reviews and Meta-Analyses Statement [18]. The protocol has been registered on Prospero (CRD42018099312).

### Study selection criteria

Research articles published in English between January 1957 and November 2021, describing studies that used quantitative preference elicitation techniques (e.g. conjoint analysis, discrete choice experiment (DCE)) to investigate preferences for RA treatment or prevention in adults aged 18 years or older were included. The start date for the search marks the publication of the first successful randomised trial of a therapeutic intervention (glucocorticoids) for RA [19]. In line with the Durand et al. review [6], articles that assessed more than one treatment attribute were included. Where multiple publications described data from a single study, the earliest publication was included, and any subsequent article was used to supplement data extraction where necessary.

Review articles, conference proceedings, abstracts, commentaries, editorials, opinion pieces and letters, qualitative studies and articles using time-trade off methods for economic evaluations were excluded. Studies assessing healthcare professionals’ preferences were also excluded unless data for patient/public preferences could be extracted separately.

### Search strategy

The following databases were searched on the 5th of November 2021 to identify potential articles for inclusion: MEDLINE, PsycINFO; EMBASE, Econlit publications; and CINAHL. Search terms were developed by the authors and received expert input from a librarian at the University of Birmingham. The MEDLINE search terms are provided in Additional Table S1 as an example, similar search terms were used when searching the other databases, only updated to the format of a particular database and restricting the search to that database only.

### Article selection

A minimum of two reviewers independently screened the titles and abstracts identified by the search strategy for potential eligibility for inclusion. Reviewers discussed any disagreements and where necessary an additional reviewer (GS) screened the abstract in question and disagreements were resolved by consensus if possible or the article in question was put forward for full-text review. Full-text review was again conducted independently by a minimum of two reviewers (GS and MF) and where there was any uncertainty about the validity of the inclusion of a source, methodological experts were consulted (JV and RD) Reference lists of all articles included were checked to identify further eligible studies.

### Assessment with the PREFS checklist

Included articles were reviewed independently by two research assistants (GM and NW) using the 5-item Purpose, Respondents, Explanation, Findings, Significance (PREFS) checklist [20]. The PREFS checklist was developed to access quality and validity across different types of treatment preference study. This checklist assesses whether (1) preference assessment is clearly defined and is the main objective(s) of the study, (2) there is the risk of a selection bias, (3) enough methodological detail is available to enable replication of the study, (4) there is a risk of bias arising from excluding data from the findings, and (5) key results and significance tests were reported. Scores for each item and an aggregate score (ranging from 0 to 5) were calculated for each included study. Where the reviewers differed in their assessment, the source was assessed by an additional reviewer (GS) and consensus reached through discussion amongst all reviewers.

### Data extraction and analysis

Data extraction from included articles was conducted by a minimum of two independent reviewers (JC, GS, research assistant). Extracted characteristics included the full reference of the source, study objective, stated preference methodology, and a description of attributes and levels and order of relative importance (See Table 1 for an overview).

**Table 1:** Extracted information

Information extracted for each source (where available)	
• Full reference of publication	
• Study objective	
• Stated preference methodology	
• Number and type of participants (patient, FDR or member of the public)	
• Participant characteristics and inclusion criteria	
• Country the study took place in	
• Description of attributes and attribute levels	
• Absolute rank order of relative importance of the attributes	
• Basis for attribute selection and presentation	
• Involvement of stakeholders in selection of attributes	

The attributes in each study were further categorised according to whether they described a **risk** (e.g. ‘Risk of serious infection’), **benefit** (e.g. ‘Likelihood of remission’ or ‘Reduction in chance of developing RA’), **treatment administration** (e.g. ‘Route of administration’), or **cost** of the treatment (e.g. ‘Personal cost to you per month not covered by insurance’). All other attributes were categorised as ‘**other**’.

## Results

### Study selection results

**Fig. 1:**
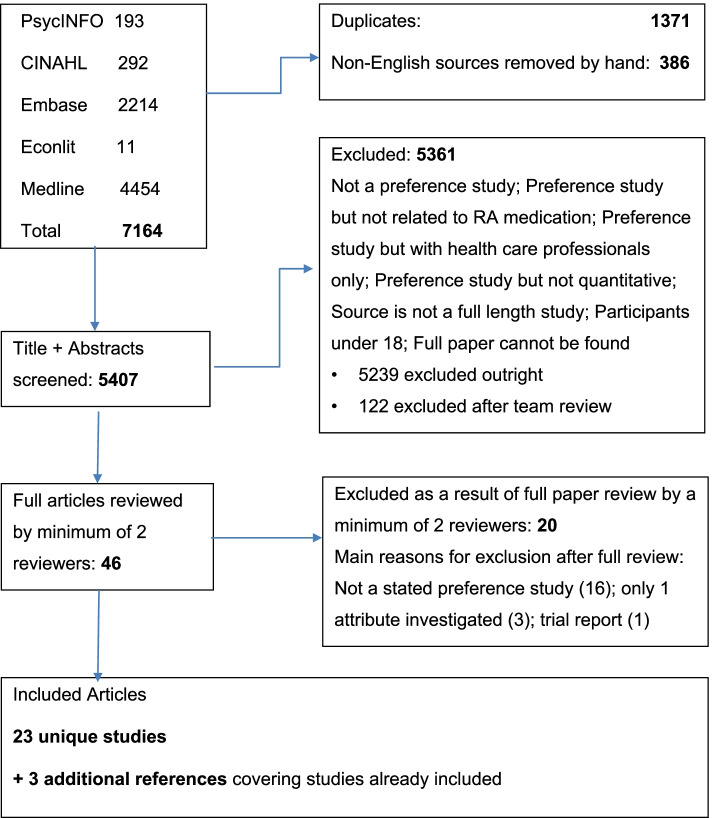
Study selection results

Twenty-three unique studies were included from the 5407 screened records (see Fig. 1). Three of these studies were described in multiple publications. Only one paper for each was included in the systematic review, with the others referenced. No studies were identified through the references of the included studies.

Table 2 summarizes the characteristics of included studies. None of the included studies predates 2004 and 20 of the 23 studies were published after 2012. The study objectives for the included studies can be found in Additional Table S2. Twenty studies were of preferences for RA treatment and three studies for RA prevention. Study participants were patients with established (*n* = 17) or newly diagnosed (*n* = 1) RA; members of the general population (*n* = 2) who were asked to imagine they had RA; first degree relatives (FDRs) of patients with RA (*n* = 2); and one study included both FDRs and RA patients. Some articles also reported data from patients with other conditions, and healthcare professionals but these were not extracted for the current review. Sample sizes varied between 85 and 2663 individuals for the RA treatment studies and between 30 and 288 individuals in the prevention studies. Studies were carried out in the USA (*n* = 9), Europe (*n* = 8), Canada (*n* = 4), Australia (1), and Argentina (*n* = 1). Studies included were DCEs (*n* = 15), conjoint analyses (*n* = 6), or Best-Worst Scaling (*n* = 2). A description of participant characteristics such as age and gender distribution and the (clinical) inclusion criteria for each study can be found in Additional Table S3.

**Table 2:** Overview included studies including PREFS assessment

Source	Method	Participants	Country	PREFS
**Studies of rheumatoid arthritis treatments**				
1) Alten et al. [21]	Best-Worst scaling	1588 RA patients	Germany	3
2) Augustovski et al. [22]	DCE	240 RA patients	Argentina	3
3) Bywall et al. [23, 24]	DCE	358 patients with RA	Sweden	4
4) Constantinescu et al. [25, 26]	Conjoint analysis	136 RA patients	USA	4
5) Díaz-Torné et al. [27]	DCE	137 RA patients	Spain	3
6) Fraenkel et al. [28]	Conjoint analysis	120 RA patients	USA	3
7) Fraenkel et al. [29]	Conjoint analysis	156 RA patients	USA	3
8) Fraenkel et al. [30]	Conjoint analysis	1273 RA patients	USA & Puerto Rico	4
9) Hazlewood et al. [31, 32]	DCE	152 Early RA patients	Canada	5
10) Ho et al. [33]	DCE	RA patients	Australia	4
11) Husni et al. [34]	DCE	510 RA Patients	USA	4
12) Louder et al. [35]	Conjoint analysis	380 RA patients	USA	5
13) Nolla et al. [36]	Conjoint analysis	165 RA patients	Spain	3
14) Ozdemir et al. [37]	DCE	463 RA patients (233 Cheap-talk and 230 Control)	USA	3
15) Poulos et al. [38]	Conjoint Analysis	836 RA patients	USA	5
16) Scalone et al. [39]	DCE	174 RA patients	Italy	3
17) Skjoldborg et al. [40]	DCE	178 RA patients (145 survey 2; 130 survey 3)	Denmark	4
18) van Heuckelum et al. [41]	DCE	325 RA patients	The Netherlands	4
19) Bansback et al. [42]	DCE	2663 General population asked to imagine they have RA	Canada	4
20) Harrison et al. [43]	DCE	733 General population asked to imagine they have RA	Canada	4
**Studies of RA prevention**				
21) Finckh et al. [44]	Best worst scaling	32 FDRs	Switzerland	4
22) Harrison et al. [45]	DCE	288 FDRs	USA	4
23) Harrison et al. [46]	DCE	30 FDRs 78 RA patients	Canada	4

### Results from the transparency assessment with the PREFS checklist

Aggregate PREFS scores are included in Table 2. Eight studies scored 3 (out of 5), twelve (including all three studies of treatments to prevent RA) scored 4, and three scored 5. Most studies failed to give information about how respondents differed from non-respondents reducing the score to 4 or less.

Twenty sources provided either a sample task or a complete survey. Only the three studies of RA prevention, and two treatment studies (one with non-patient participants) included the background information provided to participants.

### Attributes, attribute selection, and order of relative importance

There was wide variation in the number and type of attributes included, and number of attribute levels across studies. Table 3 provides an overview of the attribute selection process, whereas Table 4 provides the attributes included as well as the type of attribute and the absolute rank order of the relative importance for each study where this information is available. For two studies, the rank order of attribute importance was either not reported or could not be derived from the reported results and model [29, 41]. An overview of the attributes and attribute levels for all 23 included studies can be found in Additional Table S2.

**Table 3:** Method of attribute selection

Source	Method of attribute selection	Stakeholder involvement^a^
1 [21]	Review of existing literature and/or other sources, and qualitative research	Yes
2 [22]	Review of existing literature or other sources, expert opinion and qualitative research	Yes
3 [23, 24]	Review of existing literature or other sources, expert opinion and qualitative research	Yes
4 [25, 26]	Review of existing literature and/or other sources	No
5 [27]	Review of existing literature and/or other sources, expert opinion, and qualitative research	Yes
6 [28]	Review of existing literature and/ or other sources	No
7 [29]	Review of existing literature and/or other sources, and review of selected attributes by clinicians and stakeholders	Yes
8 [30]	Review of existing literature and/or other sources, and review of selected attributes by clinicians and stakeholders	Yes
9 [31, 32]	Expert opinion and qualitative research	Yes
10 [33]	Review of existing literature and/or other sources and qualitative research	Yes
11 [34]	Review of existing literature and/or other sources, and review of selected attributes by clinicians and stakeholders	Yes
12 [35]	Review of existing literature and/or other sources	No
13 [36]	Review of existing literature and/or other sources	No
14 [37]	Review of existing literature and/or other sources and qualitative research	Yes
15 [38]	Review of existing literature and/or other sources	RA patients were involved in pre-testing only
16 [39]	Review of existing literature and/or other sources and qualitative research	Yes
17 [40]	Review of existing literature and/or other sources	No
18 [41]	Review of existing literature or other sources, expert opinion and qualitative research	Yes
19 [42]	Review of existing literature and/or other sources and qualitative research	Yes
20 [43]	Review of existing literature and/or other sources and qualitative research	Yes
21 [44]	Review of existing literature and/or other sources and qualitative research	Yes
22 [45]	Review of existing literature and/or other sources, expert opinion and qualitative research [47]	Yes
23 [46]	Review of existing literature and/or other sources, expert opinion and qualitative research [47] (attributes included on the basis of attribute selection in source 19)	Yes

^a^Stakeholders: *RA* patients in RA treatment studies, *FDRs* in preventive treatment studies

**Table 4:** Order of relative importance

Source	Order of relative importance attributes (type of attribute)		
1 [21]	(1) Route of administration (TA); (2) combination therapy (O); (3) frequency of administration (TA); (4) possible side effects (R); and (5) time till onset drug effect (B)		
2 [22]	(1) Cost per month (C); (2) general adverse events (R); (3) frequency of administration (TA); (4) efficacy patient global assessment (B); (5) route of administration (TA); (6) local adverse events (R); and (7) serious infection (R)		
3 [23, 24]	(1) Treatment effectiveness (B); (2) severe side effects (R); (3) psychological side effects (R); (4) route of administration (TA); (5) frequency of administration (TA); (6) side effects changing appearance (R); and (7) mild side effects (R)		
4 [25, 26]	**African-American participants:** (1) Risk of cancer (R); (2) likelihood of remission (B); (3) risk of lung injury (R); (4) route of administration (TA); (5) likelihood of symptoms improving (B); (6) likelihood of arresting radiographic progression (B); (7) risk of injection reaction (R); (8) risk of tuberculosis (R); (9) risk of neurologic disease or heart failure (R); and (10) reversible adverse events (R)	**White participants:** (1) Likelihood of remission (B); (2) likelihood of arresting radiographic progression (B); (3) likelihood of symptoms improving (B); (4) risk of cancer (R); (5) risk of lung injury (R); (6) route of administration (TA); (7) risk of injection reaction (R); (8) risk of neurologic disease or heart failure (R); (9) reversible adverse events (R.); and (10) risk of tuberculosis (R)	
5 [27]	(1) Time with optimal quality of life (B); (2) mode of administration (TA); (3) onset of treatment action (B); (4) probability of severe adverse events (R); (5) monthly co-pay (C); (6) substantial Improvement in symptoms (B); and (7) Probability of mild adverse events (R)		
6 [28]	(1) Less common, but serious AE (R; separate attributes: kidney damage/liver damage/cancer/lung damage); (2) common, but reversible AE (R; separate attributes: alopecia/oral ulcers/nausea/injection reaction/rash/diarrhoea); (3) route and frequency of administration (TA); (4) drug onset (B); (5) monthly co-pay (C); (6) physician experience (O); (7) chance of benefit (B) and (8) no new bone damage at year1 (B)		
7 [29]^a^	**No order of relative importance available:** Decreased joint pain and swelling (B); ability to get around and participate in social or leisure activities outside of the house (B); slowing or stopping joint damage seen on x-rays (B); ability to work (B); risk of injection/infusion reaction (R); risk of infection (R); risk of TB (R) and risk of neurological disease (R)		
8 [30]	(1) Cost (C); (2) bothersome side effects (R); (3) very rare side effects (R); (4) onset of action (B); (5) serious infection (R); (6) route of administration (TA); and (7) amount of information available (O)		
9 [31, 32]	(1) Major symptom improvement by 6 month (B); (2) reduction in chance of serious joint damage (B); (4) route and frequency of administration (TA); (5) small risk of serious infection/possible increased risk of cancer (R); (6) stopping due to side effect by 6 months (R); (7) lung/liver reaction (O); (8) alcohol restriction (O); and (9) eye screening (O)		
10 [33]	**Reported for RA patients, AS patients and PsA patients combined**^**b**^**:**		
	**Oral** (1) Efficacy (B); (2) slowing of disease progression (B); (3) mild-moderate side effects (R); and (4) nurse support (O)	**Injection** (1) Efficacy (B); (2) slowing of disease progression (B); (3) mild-moderate side effects (R); (4) nurse support (O); and (5) serious side effects (R)	**IV** (1) Efficacy (B); (2) slowing of disease progression (B); (3) mild-moderate side effects (R); (4) frequency of administration (TA); (5) serious side effects (R); and (6) nurse support (O)
11 [34]	(1) Improvement in physical function (B); (2) reduction in pain (B); (3) reduction in number of swollen joints (B); (4) route of administration (TA); (5) risk of cancer (R); (6) monthly co-pay (C); (7) frequency of administration (TA); (8) abnormal lab results (R); and (9) risk of serious infection (R)		
12 [35]	(1) Route of administration (TA); (2) frequency of administration (TA); (3) serious adverse events (R); (4) monthly co-pay (C); (5) medication burden (O); (6) joint pain reduction (B); and (7) daily task improvement (B)		
13 [36]	(1) Pain relief and improvement in functional capacity (B); (2) risk of adverse events (R); (3) route of administration (TA); and (4) duration of effect (B)		
14 [37]	**Cheap talk sample only: (**1) Monthly co-pay (C); (2) chance the medication works well (B); (3) route of administration and frequency (TA); (4) serious infection (R); (5) onset of effect (B); and (6) duration of injection site irritation (R)		
15 [38]	(1) Immediate serious reaction (R); (2) medication working well (B); (3) frequency of administration (TA); (4) time for infusion (TA); (5) immediate mild reaction (R); and (6) route of administration (TA)		
16 [39]	(1) Frequency of reactions at the site of drug administration (R); (2) additional costs through taxes (C); (3) manner, helpfulness, efficiency and courtesy of health personnel (O); (4) generalised undesired reactions or allergic reactions (R); (5) Route and place of administration (T); and (6) frequency of administration (TA)		
17 [40]	**WTP (1000 DKK), survey 1 only: (**1) tiredness (B); (2) slightly higher risk of a minor infection (R); (3) pain level (B); (4) swollen joints (B); (5) morning stiffness (B); and (6) co-pay (C)		
18 [41]^a^	**No order of relative importance available:** Route of administration (A); frequency of administration (A); onset of action (B); risk of cancer (R); risk of liver injury (R); risk of serious infections (R); and chance of efficacy (B)		
19 [42]	(1) How many people receiving the drug are likely to feel better within 6 months (B); (2) route and duration of administration (TA); (3) life-expectancy (B)^c^; (4) minor side effects (R); (5) frequency of administration (TA); and (6) serious side effect: number of people have to stop medication (R)		
20 [43]	(1) How many people receiving the drug are likely to feel better within 6 months (B); (2) confidence in risk/benefit estimates (O); (3) serious side effect: number of people have to stop medication (R); (4) route of administration (TA); and (6) frequency of administration (TA)		
21 [44]	(1) Reduction in RA risk (B); (2) risk of SAE (R); (3) mild AE (R); and (4) mode of administration (TA)		
22 [45]	(1) Route of administration (TA); (2) reduction in RA risk (B); (3) health care professional preference (O); (4) chance of side effects (R); and (5) certainty in evidence (O)		
23 [46]	**Patients and FDR sample combined: (**1) Health care professional preference (O); (2) chance of side effects (R); (3) reduction in RA risk (B); (4) route of administration (TA); and (5) certainty in evidence (O)		

Type of attribute: risk (R), benefit (B), treatment administration (TA), cost (C), or other (O); IV intravenous infusion

^a^ Attribute order of importance not available

^b^ It was not possible to establish the order of importance for the RA patients alone, so results presented are for the whole sample (RA, AS and PsA). Further as the model used was a restricted latent class model with parameters that were non-significant in the preliminary models restricted to zero, they are not reported here as they were deemed not to impact on preferences

^c^ Life expectancy was assumed to be linear over the 4-year range in the study

Attribute selection was informed by either literature review, clinical opinion, qualitative research or a combination thereof. Fourteen out of 20 studies of RA treatment preferences (12 of the patient studies and both general population studies) reported including at least one stakeholder in the form of RA patients in the attribute selection process. All three studies of preferences for RA prevention included a qualitative phase to which FDRs contributed.

The variability of attributes and levels made comparisons across studies and between different participant groups difficult. However, there were some notable differences. The most common number of attributes according to the mode was seven. Six patient studies of RA treatment included large numbers of attributes (between 8 and 16) often describing multiple specific side effects (e.g. [25, 26, 28]). In contrast, all three prevention studies included five or fewer attributes.

All but one study [39] included at least one benefit attribute. Benefit attributes for RA treatment studies focussed on efficacy in symptom reduction and remission, except two studies [21, 30], which instead included time till onset of treatment action. Those with general public participants included the percentage of people treated that would feel better in six months. RA prevention studies included reduction in risk of developing RA.

In ten of the studies of RA treatments, risk attributes related to specific conditions, such as risk of cancer or tuberculosis. The other RA treatment studies, including those with general public participants, and the three preventive treatment studies included risk attributes as side effects described in terms of their severity (e.g. ‘minor side effects’) or the proportion of people having to discontinue treatment due to adverse events, in some cases giving examples of the type of side effects these categories might entail, but not singling out one specific side effect. One study of RA treatments referred to psychological side effects and side-effects related to physical appearance in addition to mild and serious side effects [23, 24]. Presentation of side effects as risk of specific conditions versus categories of side effect severity did not appear to affect relative importance of risk attributes.

A benefit attribute was ranked higher than a risk attribute in both of the RA treatment studies with general public participants [42, 43]. However, for the studies with RA patients a benefit attribute was relatively more important than a risk attribute in only eight of the 16 studies that assessed a benefit attribute and for which a rank ordering for relative importance was available [23, 24, 27, 31–34, 36, 37, 40]. In addition, for one study [25, 26], the rank order differed for the two samples. Whereas a sample of African-American participants placed more relative importance on a risk attribute over benefits, the sample of white participants placed more relative importance on a benefit (likelihood of remission; see also Table 3). A benefit was ranked higher than risk attributes in two of the three prevention studies [44–46].

All but two studies [29, 40] included at least one treatment administration attribute. For these treatment attributes, only the prevention studies included information on how long medication would need to be taken (i.e. 1 year). Treatment attributes mainly included frequency and route of administration either as individual attributes or as a combination. In one study, a labelled experimental design was used, with the treatment profiles indicated as ‘oral’, ‘injection’, ‘infusion’ and an opt out [33].

Nine of the 18 RA treatment studies with patient participants also included cost as an attribute, whereas none of those with general public participants did, nor did the RA prevention studies. Two prevention studies [45, 46] reported excluding a cost attribute as it was ranked as the least important attribute in the qualitative research conducted to inform attribute selection [47]. Cost attribute ranges examined across studies varied widely, which was reflected in the relative importance across studies. Moderate to large ranges of cost levels (e.g. ranging from 0 to $1000 per month or from ‘easy’ to ‘hard to afford’) were important determinants of choice (ranked first or second) in five of the nine studies. Among those studies where cost was ranked lower, smaller ranges of cost attribute levels (e.g. 0 to $50 per month) were typical.

As shown in Table 3, a number of attributes were coded as **other** including: physician experience or recommendation, the availability of nurse support, additional burden associated with the treatment in the form of regular medical tests or having to take it together with another medicine, and degree of uncertainty around benefit and risk estimates. Attributes that characterise the degree of certainty around the benefits or risk of treatment were included in two out of three prevention studies [45, 46] and one out of two RA treatment studies with general public participants [43]. Three RA treatment studies with patient participants included attributes relating to how long a treatment has been in use [28, 30, 33]. There was variation across these studies in terms of the relative importance of this kind of attributes.

## Discussion

The aim was to systematically review preference studies related to treatments for RA, including both patient and general public participants and treatments to prevent RA. This updates and extends a previous systematic review [6] by including more recently published studies and studies in non-patient populations.

The fact that preventive intervention for RA in at-risk populations is a relatively recent area of research focus, was reflected in the fact that only three preference studies for preventive treatment were identified, two of which were conducted by the same research group and all with relatively small sample sizes. The relative importance of types of attributes was variable across these three studies, suggesting that further evidence is needed in this area.

The quality of all included sources was assessed with the PREFS checklist, which rewards transparency. Transparency is desirable to allow interpretation of results and replication of studies. Eight of the 18 studies with patient participants were assigned a relatively low PREFS score (three out of five), indicating that the description of the study method and results section lacked detail. The most frequent contributor to a lower score was respondent sampling and not addressing similarity of respondents and non-respondents.

Most of the included sources used attributes typical of treatment preference studies, such as side effects, treatment efficacy, mode of administration and frequency of treatment administration. Treatment cost or co-pay was included in half of the studies with RA patients, mostly in countries without universal health care provision (e.g. USA) although there were exceptions where for example increased taxation was included as a form of co-pay (e.g. Italy [39]). Where cost was included, it was an important determinant of choice in five of the nine studies, typically where the cost ranges studied were higher.

The number and type of attributes included, and the absolute rank order of relative importance of types of attribute, varied across studies. Studies of preferences for RA treatment tended to include more attributes than studies looking at preferences for preventive treatment.

When comparing the relative ranking of benefits and risks for treatment studies and prevention studies, it was found that a benefit attribute was ranked higher than a risk attribute over the ranges tested for two out of three prevention studies and in both studies with the general public. In contrast, for the studies of RA treatments, a benefit attribute was relatively more important than a risk attribute in only half of the studies. Due to the smaller number of non-patient studies, and methodological heterogeneity across these studies, it is difficult to draw conclusions about differences in the relative preferences of patient and non-patient participants. It is possible that different levels of first-hand experience with a disease or personal experience of treatment side effects could influence preference results [48]. Direct comparisons between different samples with different levels of disease proximity are now needed, especially in the context of studies of preferences for preventive treatments. Where study participants do not have the disease in question, preferences could be especially likely to vary according to participants’ illness perceptions or beliefs about medication. Two studies identified in this review included a measure of patients’ beliefs about medication, but none of the studies of RA prevention did. Further research is needed to elucidate the role of such psychological constructs in preference heterogeneity [49].

About half of the studies of patients’ preferences for RA treatment identified in this review included specific side effects as separate risk attributes, such as the risk of (a specific) cancer or the risk of serious infection. Whereas the remaining studies used more general terms such as “serious side effect”, some would include examples to illustrate what might constitute such a side effect. Specificity of the risk description does not appear to impact on the rank ordering of attributes in terms of relative importance. However, where risks of multiple conditions are included as separate attributes there is potential for level overlap and decreased ability of participants to make an informed choice. Risk attributes in the studies identified were typically described in terms of their severity (e.g. ‘mild side effect’), but one source included further categories of side-effects, such as those affecting an individual’s appearance or mental health which had varying importance for preferences for different sub-groups of participants.

Five studies varied the degree of confidence around treatment efficacy and safety, or degree of approval of their healthcare professional [28, 30, 43, 45, 46]. This approach was more frequent, and more likely to have an important impact on preferences in studies with non-patient participants. In clinical settings, people would not usually be asked to consider a treatment for which there is insufficient evidence and there may be potential for a related attribute to dominate preferences. However, this is an important consideration for clinical trial design, where consideration and communication of risks and benefits in the context of limited evidence is essential.

The current review was comprehensive and methodologically rigorous. The study protocol benefited from the extensive input from a multidisciplinary team including clinical and methodological expertise and patient research partners. There are limitations to the ability to compare preference studies with different objectives, populations, formats, and attributes. Further work to explore the impact of differences in attribute presentation and wording is needed. Further, as noted in the previous review of the preference literature in RA [50], some studies were fully or partially industry-funded (e.g. [22, 33, 35, 36, 38], which may have introduced a bias towards the inclusion of, and emphasis on certain attributes (e.g. mode of administration) and the exclusion of others.

The studies identified in this review all took place between 2004 and 2021, with most of them taking place after 2012. Whilst this is a relatively narrow window of time, it should be noted that the availability of certain categories of drugs and of information about treatment effectiveness and safety varies over time, and limits the extent to which temporally separated studies can be compared. A final limitation of this study is that the search strategy was restricted to articles published in the English language only, which may have resulted in the exclusion of relevant articles published in other languages.

This review has highlighted a need for further studies of preferences of at-risk populations for treatments to prevent RA. The three studies in this context identified included very small samples of FDRs of patients with a confirmed diagnosis of RA or self-reported FDRs. Many public misperceptions exist around the nature and symptoms of RA [51, 52] and RA is often confused with osteoarthritis, raising questions around sample integrity when participants are self-reported FDRs. Further studies with FDRs recruited through patients of rheumatology clinics with a confirmed diagnosis are needed, though such recruitment is challenging and resource intensive [53]. As preference studies of this kind typically ask participants to imagine a scenario where they have been identified as being at high risk of developing RA, it is possible that the preferences of the general population are equivalent to those of at-risk groups and more readily available to study. Direct comparisons are needed to address this important issue. The elicitation of public preferences is also in line with publicly funded health system requirements to value healthcare interventions based on the preferences of the general population [54].

It is further important to note that most ongoing prevention trials focus on symptomatic at-risk groups (e.g. people with clinically suspect arthralgia [55]) and there is a need for future preference studies to quantify the preferences of this group, as none were identified in this review. It is likely that such studies could include benefit attributes relating to reduction in current symptoms (e.g. joint pain; fatigue) in addition to risk reduction benefits.

## Conclusion

Most stated preference studies relating to RA focus on the treatment of established disease. These find that treatment attributes, such as efficacy, safety, route/mode of administration and treatment cost are important determinants of choice. There are few studies that examine preferences of at-risk individuals for treatments to prevent RA. Given the increasing research focus on RA prediction and prevention [56], there is a need for further preference studies in this context to inform future management of RA and the design of prevention trials. Further research is also needed to assess the impact of variations in attribute presentation, predictors of preference heterogeneity and variation between populations, in the context of both disease treatment and prevention.

## Supplementary Information

**Additional file 1: Table S1.** (Medline search terms) contains the MEDLINE search terms as an example of the database searches conducted (Systematic review of quantitative preference studies of RA treatments)

**Additional file 2: Table S2.** (Study objectives, attributes and attribute levels for included studies) contains the study objectives of the included material and an overview of attribute levels (Systematic review of quantitative preference studies of RA treatments)

**Additional file 3: Table S3.** (Participant characteristics and inclusion criteria) contains the participants characteristics and (clinical) inclusion criteria (Systematic review of quantitative preference studies of RA treatments)

## Data Availability

All data generated or analysed during this study are included in this published article [and its supplementary information files].

## Abbreviations

DCE: Discrete choice experiment
EFPIA: European Federation of Pharmaceutical Industries and Associations
EULAR: European League Against Rheumatism
FDR: First degree relative
IV: Intravenous infusion
PREFER: Patient Preferences in Benefit-Risk Assessments during the Drug Life Cycle
PREFS: Purpose, Respondents, Explanation, Findings, Significance Checklist
RA: Rheumatoid arthritis
