# Supplementary material for: Systematic review of quantitative preference studies of treatments for rheumatoid arthritis among patients and at-risk populations
Source: Arthritis Res Ther. 2022 Feb 22;24:55. doi: 10.1186/s13075-021-02707-4 (PMC8862509; doi:10.1186/s13075-021-02707-4)
Supplement: Supplementary file 1 — Additional file 1: Table S1. (Medline search terms) contains the MEDLINE search terms as an example of the database searches conducted (Systematic review of quantitative preference studies of RA treatments) [file 13075_2021_2707_MOESM1_ESM.docx]

Additional Table S1. Search terms by database

| **Medline** |
| --- |
| 1. Rheumatoid Arthritis.mp. or Arthritis, Rheumatoid/  2. PATIENT PREFERENCE/ or Preference*.mp.  3. Choice*.mp.  4. decision making/ or decision making.mp.  5. choice behavior/ or choice behavior.mp. or choice behaviour.mp.  6. Accept*.mp.  7. Utilit*.mp.  8. Desirab*.mp.  9. therapeutics/ or biological therapy/ or drug therapy/ or remission induction/  10. therap*.mp.  11. treatment*.mp.  12. MEDICINE/ or PREVENTIVE MEDICINE/  13. medicine*.mp.  14. Pharmaceutical Preparations/  15. Drug*.mp.  16. EARLY MEDICAL INTERVENTION/mt, ut [Methods, Utilization]  17. intervention*.mp.  18. Medication*.mp.  19. Pharmaceutical*.mp.  20. 2 or 3 or 4 or 5 or 6 or 7 or 8  21. 9 or 10 or 11 or 12 or 13 or 14 or 15 or 16 or 17 or 18 or 19  22. 1 and 20 and 21  23. limit 22 to yr="1957 -Current" |
