# Supplementary material for: Systematic review of quantitative preference studies of treatments for rheumatoid arthritis among patients and at-risk populations
Source: Arthritis Res Ther. 2022 Feb 22;24:55. doi: 10.1186/s13075-021-02707-4 (PMC8862509; doi:10.1186/s13075-021-02707-4)
Supplement: Supplementary file 2 — Additional file 2: Table S2. (Study objectives, attributes and attribute levels for included studies) contains the study objectives of the included material and an overview of attribute levels (Systematic review of quantitative preference studies of RA treatments) [file 13075_2021_2707_MOESM2_ESM.docx]

Additional table S2. Study objectives, attributes and attribute levels for included studies

| **Source** | **Study objective** | **Attributes and attribute levels** |
| --- | --- | --- |
| 1) Alten et al., 2016(1) | To assess the importance of oral administration for patient preferences among other treatment characteristics (DMARDs) | 1. Route of administration (Subcutaneous; IV; Oral) 2. Frequency of administration (2 x day; 1x every 1–2 weeks; 1x every 4–8 weeks; 1x every 6–12 months) 3. Combination therapy (yes MTX 1 x week; no, not necessary) 4. Possible side effects (Infections; Allergic reactions; Deterioration of lab. values) 5. Time till onset drug effect (up to 1 month; 1-3 months) |
| 2) Augustovski et al., 2013(2) | To assess patients’ preferences for RA treatments with biologic agents. | 1. Route of administration (Oral; Subcutaneous; IV) 2. Frequency of administration (Every 10 months; Monthly; Weekly; Daily) 3. Cost per month (0 Argentine pesos; 500; 1500) 4. General adverse events (No risk; 10/100 patients; 30/100 patients) 5. Local adverse events (No risk; 15/ 100 patients; 40 / 100 patients) 6. Serious infection (1/100 patients; 5/100 patients) 7. Efficacy patient global assessment (Δ 40mm; Δ 30mm; Δ 20mm) |
| 3) Bywall et al.,2021(3); 2020 (4) | To assess patients’ preferences for second-line therapies | 1. Route of administration (Oral; Subcutaneous; IV) 2. Frequency of use (Daily, weekly, Monthly) 3. Mild short-term side effects (1 in 10, 1 in 100, 1 in 1000) 4. Side effects changing appearance (1 in 10, 1 in 100, 1 in 1000) 5. Probability of psychological side effects (1 in 10, 1 in 100, 1 in 1000) 6. Probability of severe side effects that requires hospitalisation such as severe infections or allergic reactions (1 in 10, 1 in 100, 1 in 1000) 7. Effectiveness (30% improvement, 50%, 70%) |
| 4) Constantinescu et al., 2009(5, 6) | To assess whether African American and white RA patients differ in how they evaluate the specific risks and benefits related to medications. | 1. Route of administration (Weekly oral; Self-injection 1x every 1-2 weeks; IV every 6-8 weeks) 2. Risk of cancer (No increased risk; 1/1,000 patients) 3. Risk of lung injury (No risk; risk of lung injury 2 /100 patients or liver injury 1 / 1,000 patients) 4. Risk of tuberculosis (No increased risk; 1/10,000 patients) 5. Risk of neurologic disease or heart failure (No increased risk; 1/10,000 patients) 6. Risk of injection reaction (No injection reactions; 30 / 100 patients get a rash or local burning at the site of injection; 3 of/100 patients will get a reaction during the infusion (headache, nausea, fever) 7. Reversible adverse events (No increased risk of nausea, dizziness, or unusual tiredness; 10 /100 people.) 8. Likelihood of remission (45 /100; 25 /100; 15 /100) 9. Likelihood of symptoms improving (70/ 100 patients; 50/100 patients; 40/ 100 patients) 10. Likelihood of arresting radiographic progression 80 / 100 patients; in 50 / 100 patients; in 30 / 100 patients. |
| 5) Díaz-Torné et al., 2020(7) | To assess patient and rheumatologist preferences for RA treatment attributes in Spain and to evaluate their attitude towards shared decision-making (SDM). | 1. Mode of administration (Daily oral; Weekly subcutaneous; Monthly subcutaneous; Monthly IV) 2. Probability of severe adverse events (1/100 patients; 5/100) 3. Probability of mild adverse events (10/ 100 patients; 30/100) 4. Monthly co-pay (0; 10; 20; 50 Euro) 5. Substantial Improvement in symptoms (9/10 patients; 5/10; 3/10) 6. Onset of treatment action (7 days; 15 days; 1 month; 3 month) 7. Time with optimal Quality of Life (10 years;7; 5; 2) |
| 6) Fraenkel et al.,2004(8) | To assess patients’ treatment preferences for DMARDs with varying risk profiles. | 1. Route and frequency of administration (Weekly oral; Twice weekly subcutaneous self-injection; Weekly intramuscular injection given by nurse in clinic) 2. Less common, but serious AE (entered as separate attributes: kidney damage 0%; 1% / liver damage 0%; 0.1% / cancer 0%; theoretical but unproven increased risk/ lung damage 0%; 0.1%; 1%) 3. Common, but reversible AE (entered as separate attributes: alopecia 0%; 10%/ oral ulcers 0%; 10%/ nausea 0%; 10%; 30%/ injection reaction 0%; 40%/ rash 0%; 40%/ diarrhoea 0%; 10%; 30%) 4. Physician experience (Drug used to treat arthritis for more than 20 years; New drug with unknown long term safety profile) 5. Monthly co-pay (0; $5 per month; $15; $30) 6. Drug onset (2 weeks; 4; 8) 7. Chance of benefit (75%; 60%; 45%) 8. No new bone damage at year1 (75%; 60%) |
| 7) Fraenkel et al.,2015(9) | To assess the influence of subjective numeracy on RA patient preference for the status quo and to determine whether age modifies this relationship. | 1. Decreased joint pain and swelling (70 / 100 people feel much better; 40/ 100 people; no chance) 2. Ability to get around and participate in social or leisure activities outside of the house (70 / 100 people can get around much easier; 40/ 100 people; no change) 3. Slowing or stopping joint damage seen on x-rays (80/ 100 people have no further bone damage seen on x-rays; 30 /100 people; No change) 4. Ability to work (80/100 people are able to keep working; 60/100; no change) 5. Risk of injection/infusion reaction (No risk of an injection reaction; 3 / 100 people get an infusion reaction; 20 /100 people get a rash or burning at the injection site) 6. Risk of infection (No risk of infection; 20 /100 people get bronchitis or sinusitis; 3/ 100 people get a serious infection) 7. Risk of TB (No increased risk; 1 in 10,000 people; 5 in 10,000 people) 8. Risk of neurological disease ( No increased risk; Extremely rare risk of a neurological disease like MS; Extremely rare risk of a neurological disease that usually causes death) |
| 8) Fraenkel et al.,2018(10) | To develop preference phenotypes to facilitate shared decision-making at the point of care for patients failing methotrexate monotherapy. | 1. Route of administration (Oral; Injection; Infusion) 2. Bothersome side effects (0%; 10%; 30%) 3. Very rare side effects (Stomach or intestinal tear 0.2%; Neurological disease like multiple sclerosis 0.05%; Permanent eye problems 0.3%; Life-threatening brain infection 0.005%) 4. Serious infection (1%; 3%; 5%) 5. Cost (Easy to afford; Somewhat affordable; Hard to afford) 6. Amount of information available (A lot, on the market for 27 years; Some, on the market for 10 years; A little, on the market for 3 years) 7. Onset of action (2 weeks; 6, 12) |
| 9) Hazlewood et al., 2016(11); 2018(12) | To assess the preferences of patients with early RA for DMARDs | 1. Route and frequency of administration(Daily oral; Weekly oral; Weekly oral; Weekly injections; Weekly oral and daily oral (two pills); Weekly oral and weekly injection; Weekly oral and IV every 8 weeks at clinic; Weekly oral and daily oral(six pills)) 2. Small risk of serious infection / possible increased risk of cancer (yes/no) 3. Stopping due to side effect by 6 months (2/100 people; 10/100 people; 20/100 people) 4. Major symptom improvement by 6 month (30/100 people; 50/100; 70/100) 5. Reduction in chance of serious joint damage (2/100; 15/100 30/100 6. Lung/liver reaction (need for regular blood work; no need) 7. Alcohol restriction (yes; no) 8. Eye screening (yes; no) |
| 10) Ho et al., 2020(13) | To assess patients’ preferences for treatments in RA, IA, AS and PsA. | Three treatment profiles labelled as ‘oral’, ‘injection’, ‘infusion’   1. Clinical efficacy; 20% improvement in pain and mobility, 50%, 70%). 2. Slowing of disease progression (Does not slow disease progression, Slows disease progression, Stops disease progression) 3. Mild- moderate side effects (varies per type of administration, between 0% and 50%) 4. Risk of severe side effects (<1% chance, 1 to 5% chance). 5. Frequency of administration (varies per type of administration: from 2 a day to once every 6 months) 6. Real-world product evidence ( from ‘less than 3 years’ to ‘7 years or more’) 7. Management of related conditions; (Treatment approved only in arthritis related conditions, Treatment approved to manage other auto-immune conditions) 8. Availability of a patient support programme (varies per type of admin mode: No support service, Nurse support phone line, other support programmes and training) |
| 11) Husni et al., 2017(14) | To assess patients’ willingness to accept treatment risks to achieve improved physical function and disease control. | 1. Frequency of administration (Daily; Every two weeks; Monthly) 2. Route of administration (Oral; Subcutaneous; IV) 3. Risk of cancer (0%; 1%; 2%) 4. Abnormal lab results (10%; 20%; 30%) 5. Risk of serious infection (0%; 2%; 4%) 6. Monthly co-pay ($0; $50; $100) 7. Improvement in physical function (0%; 40%; 60%) 8. Reduction in pain (0%; 25%; 50%; 75%) 9. Reduction in number of swollen joints (0%; 25%; 50%; 75%) |
| 12) Louder et al., 2016(15) | To assess patients’ preferences for RA treatments | 1. Route of administration (Oral; Self-injection; IV) 2. Frequency of administration (Twice daily; Weekly; Every other week; Once every 8 weeks) 3. Serious adverse events (4/100 people; 6/100; 8/100) 4. Monthly co-pay ($25; $50; $75) 5. Medication burden (have to take with another medicine; not necessary) 6. Joint pain reduction (50/100 people; 52/100; 54/100; 58/100) 7. Daily task improvement (32%; 33%; 34%; 36%) |
| 13) Nolla et al., 2016(16) | To define importance values assigned to attributes of biological agents (BAs) by Spanish patients with rheumatic diseases and rheumatologists. | 1. Route of administration (Subcutaneous self-injection; IV by health care professional at hospital) 2. Risk of adverse events (High; low) 3. Pain relief and improvement in functional capacity (yes; no) 4. Duration of effect (time until perceiving the need for a new dose: 1 week; 2; 4; 8) |
| 14) Ozdemir et al., 2009(17) | To assess the effect of a split-sample, cheap-talk experiment on patients’ preferences for RA treatments in an SC survey. | 1. Route of administration and frequency ( weekly self-injection; Self-injection every 2 weeks; Self-injection every 4 weeks; IV every 8 weeks at doctor's office or clinic) 2. Serious infection (0%; 5%) 3. Duration of injection site irritation (15min; 1 hour; 3 hours) 4. Monthly co-pay ($50; $150; $300; $600; $1000) 5. Chance the medication works well (25%, 50, 75, 100) 6. Onset of effect (1 week; 2; 4; 10) |
| 15) Poulos et al., 2014(18) | To quantify the rate at which RA patients are willing to trade off between the time required to administer treatment and treatment frequency. | 1. Frequency of administration (2 weekly treatments; 1 treatment every two weeks ;1 monthly treatment; 2 treatments 2 weeks apart every 6 months) 2. Time for infusion (no time; 30 min; 1 hour; 2 hours; 4 hours) 3. Route of administration (Self-injection; IV at doctor's office or clinic) 4. Immediate serious reaction (1%; 10%; 25%) 5. Immediate mild reaction (1%; 10%; 25%) 6. Medication working well (75% patients; 60%; 40%) |
| 16) Scalone et al.,2018 (19) | To estimate preferences in relevant treatment characteristics evaluated by different groups involved in the management of patients with rheumatic diseases. | 1. Mode and place of administration (Subcutaneous self-injection with pen at home; Subcutaneous self-injection with a syringe at home; IV at an infusion center close to home; IV at the rheumatology center) 2. Frequency of administration (1x every 7-15 days; 1x every 1-2 months; 1x every 6 months) 3. Frequency of reactions at the site of drug administration (3 times or less per year; 4 or more times) 4. Generalised undesired reactions or allergic reactions (mild vs serious) 5. Additional costs through taxes (0 euro; 130 euro; 260 euro) 6. Manner, helpfulness, efficiency and courtesy of health personnel (Unsatisfactory for the patient; Fairly satisfactory; Very satisfactory) |
| 17) Skjoldborg et al., 2009(20) | To investigate the issue of conjoint reliability over time. | 1. Slightly higher risk of a minor infection (yes; no) 2. Co-pay (0; 50; 100; 200; 450; 575; 800; 900; 1075; 1150;1250; 1500; 2150; 2300; 2500; 3000; 4300; 5000 DKK) 3. Tiredness (reduced; unchanged) 4. Pain level (0; 2; 4;6; 8; 10) 5. Swollen joints (0; 5; 10; 15; 20; 25) 6. Morning Stiffness (0; 5; 30; 60; 90; 120 min) |
| 18) van Heuckelum, et al., 2019(21) | To identify subgroups of RA patients based on their shared preferences toward DMARDs, and to identify factors associated with subgroup membership | 1. Route of administration (Oral, injection, IV) 2. Frequency of administration (Monthly, Weekly, Daily) 3. Onset of action (One week, Six weeks, 12 weeks) 4. Risk of cancer (No risk, 0.1%, 0.5%) 5. Risk of liver injury (No risk, 0.1%, 1.0%) 6. Risk of serious infections (No risk, 0.1%, 1.0%) 7. Chance of efficacy (80%, 60%, 40%) |
| 19) Bansback et al., 2016(22) | To assess how 2 methods for conveying imprecision in risk influence people’s treatment decisions. | 1. Frequency of administration (Twice daily; Once weekly; Every 8 weeks) 2. Route and duration of administration (Oral, few minutes; Subcutaneous self-injection, few minutes; IV by a physician/nurse at an office/hospital, 3-4 hours) 3. Serious side effect: number of people have to stop medication (5/100; 10/ 100; 15/100) 4. Minor side effects (5/100 people; 18/100; 30/100) 5. Life-expectancy ( 6 more years; 8 more years; 10 more years) 6. How many people receiving the drug are likely to feel better within 6 months (40/100; 55/100; 70/100) 7. Imprecision (No imprecision; quantitative imprecision; qualitative imprecision). |
| 20) Harrison et al.,2015(23) | To assess the value society places on aspects of RA treatment to inform policymaking. | 1. Route of administration (Oral, few minutes; Subcutaneous self-injection, few minutes; IV by a physician/nurse at an office/hospital, 3-4 hours) 2. Frequency of administration (Twice daily; Once weekly; Every 8 weeks) 3. Minor side effects (5/100 people; 18/100; 30/100) 4. Serious side effect: number of people have to stop medication (5/100; 10/ 100; 15/100) 5. How many people receiving the drug are likely to feel better within 6 months (40/100; 55/100; 70/100) 6. Confidence in risk/ benefit estimates (limited; moderate; very confident) |
| 21) Finckh et al., 2016(24) | To assess participants’ perception of risks and benefits and willingness to undergo preventive therapy. | 1. Mode of administration (Daily oral for 1 year; Subcutaneous, every 2 weeks for 1 year; IV 2 x, 15 days apart) 2. Risk of SAE (<1%; 5%; 10%) 3. Mild AE (10%; 20%; 40%) 4. Reduction in RA risk (80%; 20%; only delay in RA development) |
| 22) Harrison et al., 2019(25) | To understand preferences for and estimate the likely uptake of preventive treatments | 1. Mode of administration (IV 2 x, 15 days apart; Weekly Injection for one year; Daily oral for one year) 2. Chance of side effects (Common minor side effect, reversible/ Uncommon serious side effect, not reversible; Common minor side effect, reversible/ Very rare serious side effect, reversible; Common minor side effect, reversible) 3. Health care professional preference (Does not prefer, Indifferent; Prefers) 4. Certainty in evidence (very little; limited; moderate) 5. Reduction in RA risk from 60/100 to (44/100; 34/100; 24/100) |
| 23) Harrison et al., 2020(26) | To understand preferences for and estimate the likely uptake of preventive treatments, and compare preferences of patients/ FDRs and rheumatologists for preventive treatments for RA, | 1. Mode of administration (IV 2 x, 15 days apart; Weekly Injection for one year; Daily oral for one year) 2. Chance of side effects (Common minor side effect, reversible/ Uncommon serious side effect, not reversible; Common minor side effect, reversible/ Very rare serious side effect, reversible; Common minor side effect, reversible) 3. Health care professional preference (Does not prefer, Indifferent; Prefers) 4. Certainty in evidence (very little; limited; moderate) 5. Reduction in RA risk from 60/100 to (44/100; 34/100; 24/100) |

1. Alten R, Kruger K, Rellecke J, Schiffner-Rohe J, Behmer O, Schiffhorst G, et al. Examining patient preferences in the treatment of rheumatoid arthritis using a discrete-choice approach. Patient Prefer Adherence. 2016;10:2217-28.

2. Augustovski F, Beratarrechea A, Irazola V, Rubinstein F, Tesolin P, Gonzalez J, et al. Patient preferences for biologic agents in rheumatoid arthritis: a discrete-choice experiment. Value Health. 2013;16(2):385-93.

3. Bywall KS, Veldwijk J, Hansson MG, Baecklund E, Raza K, Falahee M, et al. Does being exposed to an educational tool influence patient preferences? The influence of an educational tool on patient preferences assessed by a discrete choice experiment. Patient Educ Couns. 2021;104(10):2577-85.

4. Bywall KS, Kihlbom U, Hansson M, Falahee M, Raza K, Baecklund E, et al. Patient preferences on rheumatoid arthritis second-line treatment: a discrete choice experiment of Swedish patients. Arthritis Res Ther. 2020;22(1):288.

5. Constantinescu F, Goucher S, Weinstein A, Smith W, Fraenkel L. Understanding why rheumatoid arthritis patient treatment preferences differ by race. Arthritis Rheum. 2009;61(4):413-8.

6. Constantinescu F, Goucher S, Weinstein A, Fraenkel L. Racial disparities in treatment preferences for rheumatoid arthritis. Med Care. 2009;47(3):350-5.

7. Díaz-Torné C, Urruticoechea-Arana A, Ivorra-Cortés J, Díaz S, Dilla T, Sacristán JA, et al. What Matters Most to Patients and Rheumatologists? A Discrete Choice Experiment in Rheumatoid Arthritis. Advances in Therapy. 2020;37(4):1479-95.

8. Fraenkel L, Bogardus ST, Concato J, Felson DT, Wittink DR. Patient preferences for treatment of rheumatoid arthritis. Ann Rheum Dis. 2004;63(11):1372-8.

9. Fraenkel L, Cunningham M, Peters E. Subjective numeracy and preference to stay with the status quo. Med Decis Making. 2015;35(1):6-11.

10. Fraenkel L, Nowell WB, Michel G, Wiedmeyer C. Preference phenotypes to facilitate shared decision-making in rheumatoid arthritis. Ann Rheum Dis. 2018;77(5):678-83.

11. Hazlewood GS, Bombardier C, Tomlinson G, Thorne C, Bykerk VP, Thompson A, et al. Treatment preferences of patients with early rheumatoid arthritis: a discrete-choice experiment. Rheumatology (Oxford). 2016;55(11):1959-68.

12. Hazlewood GS, Bombardier C, Tomlinson G, Marshall D. A Bayesian model that jointly considers comparative effectiveness research and patients' preferences may help inform GRADE recommendations: an application to rheumatoid arthritis treatment recommendations. J Clin Epidemiol. 2018;93:56-65.

13. Ho KA, Acar M, Puig A, Hutas G, Fifer S. What do Australian patients with inflammatory arthritis value in treatment? A discrete choice experiment. Clin Rheumatol. 2020;39(4):1077-89.

14. Husni ME, Betts KA, Griffith J, Song Y, Ganguli A. Benefit-risk trade-offs for treatment decisions in moderate-to-severe rheumatoid arthritis: focus on the patient perspective. Rheumatol Int. 2017;37(9):1423-34.

15. Louder AM, Singh A, Saverno K, Cappelleri JC, Aten AJ, Koenig AS, et al. Patient Preferences Regarding Rheumatoid Arthritis Therapies: A Conjoint Analysis. Am. 2016;9(2):84-93.

16. Nolla JM, Rodriguez M, Martin-Mola E, Raya E, Ibero I, Nocea G, et al. Patients' and rheumatologists' preferences for the attributes of biological agents used in the treatment of rheumatic diseases in Spain. Patient Prefer Adherence. 2016;10:1101-13.

17. Ozdemir S, Johnson FR, Hauber AB. Hypothetical bias, cheap talk, and stated willingness to pay for health care. J Health Econ. 2009;28(4):894-901.

18. Poulos C, Hauber AB, Gonzalez JM, Turpcu A. Patients' willingness to trade off between the duration and frequency of rheumatoid arthritis treatments. Arthritis Care Res (Hoboken). 2014;66(7):1008-15.

19. Scalone L, Sarzi-Puttini P, Sinigaglia L, Montecucco C, Giacomelli R, Lapadula G, et al. Patients', physicians', nurses', and pharmacists' preferences on the characteristics of biologic agents used in the treatment of rheumatic diseases. Patient Prefer Adherence. 2018;12:2153-68.

20. Skjoldborg US, Lauridsen J, Junker P. Reliability of the discrete choice experiment at the input and output level in patients with rheumatoid arthritis. Value Health. 2009;12(1):153-8.

21. van Heuckelum M, Mathijssen EGE, Vervloet M, Boonen A, Hebing RCF, Pasma A, et al. Preferences of patients with rheumatoid arthritis regarding disease-modifying antirheumatic drugs: A discrete choice experiment. Patient Preference and Adherence. 2019;13:1199-211.

22. Bansback N, Harrison M, Marra C. Does Introducing Imprecision around Probabilities for Benefit and Harm Influence the Way People Value Treatments? Med Decis Making. 2016;36(4):490-502.

23. Harrison M, Marra C, Shojania K, Bansback N. Societal preferences for rheumatoid arthritis treatments: evidence from a discrete choice experiment. Rheumatology (Oxford). 2015;54(10):1816-25.

24. Finckh A, Escher M, Liang MH, Bansback N. Preventive Treatments for Rheumatoid Arthritis: Issues Regarding Patient Preferences. Current Rheumatology Reports. 2016;18(8):51.

25. Harrison M, Spooner L, Bansback N, Milbers K, Koehn C, Shojania K, et al. Preventing rheumatoid arthritis: Preferences for and predicted uptake of preventive treatments among high risk individuals. PLoS One. 2019;14(4):e0216075-e.

26. Harrison M, Bansback N, Aguiar M, Koehn C, Shojania K, Finckh A, et al. Preferences for treatments to prevent rheumatoid arthritis in Canada and the influence of shared decision-making. Clinical Rheumatology. 2020.
