# Supplementary material for: Systematic review of quantitative preference studies of treatments for rheumatoid arthritis among patients and at-risk populations
Source: Arthritis Res Ther. 2022 Feb 22;24:55. doi: 10.1186/s13075-021-02707-4 (PMC8862509; doi:10.1186/s13075-021-02707-4)
Supplement: Supplementary file 3 — Additional file 3: Table S3. (Participant characteristics and inclusion criteria) contains the participants characteristics and (clinical) inclusion criteria (Systematic review of quantitative preference studies of RA treatments) [file 13075_2021_2707_MOESM3_ESM.docx]

Additional table S3. Participant characteristics and inclusion criteria

| **Studies of rheumatoid arthritis treatments** | | |
| --- | --- | --- |
| **Source** | **Participant characteristics** | **Inclusion criteria** |
| 1) Alten et al., 2016(25) | 1588 RA patients: 74% female; 45% = 50–64 years of age; 55% < 10 years of disease duration; 63% mild-to-moderate disease activity; 54% receiving injectable DMARD, 57% receiving some injectable. | Formally diagnosed and under care of rheumatologist. Treated with at least one DMARD |
| 2) Augustovski et al., 2013(49) | 240 RA patients: 86% female; Mean age = 56.26 years; Median disease duration = 9 years (IQR= 5–17); Median HAQ score = 0.5 (IQR= 0–1.225); Median Clinical disease activity index = 7.5 (IQR= 3.5–16). | Aged ≥ 18 years; Formally diagnosed with RA (min 6 months) and under care of rheumatologist; Treated with at least one DMARD; Naïve to biologic agents. |
| 3) Bywall et al., 2020 (50); 2021(51); | 358 patients with RA; 77% female; 46% = 45-64 years of age; 50% < 10 years of disease duration; | Aged 18–80 years; Established RA diagnosis, and the ability to understand and answer the questions. |
| 4) Constantinescu et al., 2009(22, 23) | 136 RA patients; 67 African American, 69 White; 83% female; Mean age = 55 years (range 22-84); Mean HAQ score African American sample = 1.4 (0–2.6), White= 0.91 (0–2.4); Mean disease duration African American = 7 years (0.2–32), White = 8 years (0.1–49) | Formally diagnosed and under care of rheumatologist; Positive serum test for at least one of the RA-associated autoantibodies (rheumatoid factor or anti-cyclic citrullinated peptide); Self-identified as African American or White, able to read and write English. |
| 5) Díaz-Torné et al., 2020(29) | 137 RA patients; Mean age = 47.5 years(SD = 10.7); 84% female; Mean disease duration 14.2 years (SD = 11.8) | Aged ≥ 18 years; Treated with at least one DMARD over the last 12 months |
| 6) Fraenkel et al.,2004(21) | 120 RA patients; mean age = 70 years (SD= 12); 76% female; mean disease duration = 8 years (SD = 5); 60% currently using a DMARD | Seen a rheumatologist for RA within last 12 months. |
| 7) Fraenkel et al.,2015(20) | 156 RA patients; mean age = 58.8 years (SD = 12.9); 85.3% female; Median disease duration = 9 years (IQR = 15); Median DAS = 14.8 (IQR =7.8) | Aged ≥ 18 years; Seen a rheumatologist for their RA min 2 times within last 12 months; Treated with at least one DMARD. Pain of at least “3” on an 11-point numeric rating scale. |
| 8) Fraenkel et al.,2018(26) | 1273 RA patients; mean age= 50.7 (SD= 11.7); 89.6% female. 24% Hispanic; Mean patient global score = 4.6 (SD = 2.3) | Aged ≥ 18 years (21 in Puerto Rico); Speak English or Spanish; Live in the USA or Puerto Rico; Report having a diagnosis of RA made by a physician; Treated with at least one DMARD and/or a biologic or JAK inhibitor. |
| 9) Hazlewood et al., 2016(31); 2018(30) | 152 Early RA patients; mean age = 52.5 (SD = 13.9); 63% female; Mean disease duration = 7.8 months (SD= 8.4); Pain VAS= 3.2 (SD = 2.6); Mean Patient global VAS = 3.0 (SD= 2.5); Mean mHAQ= 0.35 (SD= 0.48); | Patients with early RA (<2 years since diagnosis by a rheumatologist) |
| 10) Ho et al., 2020(52) | 85 RA patients; 37.65% = 41–60 years of age; 69.4% female; Mean disease duration = 8.3 years (SD= 6.6); 28.24% bDMARD-experienced ; 10.59%bDMARD-qualified; 15.29% bDMARD-naïve on opioids only; 45.88% bDMARD-naïve on other treatments (not opioids); Mean AIMS2 Standardised physical score= 2.74 (SD= 1.84); Mean AIMS2 Standardised symptom score = 4.74 (SD= 2.61) | Aged ≥ 18 years; Diagnosis of RA. Either ‘biologic or targeted synthetic DMARD-naïve’; ‘biologic or targeted synthetic DMARD-qualified’; or ‘biologic or targeted synthetic DMARD-experienced’ |
| 11) Husni et al., 2017(32) | 510 RA Patients; Mean age = 56.4 (SD= 13.8); 64.7% female; 43.1% RA > 10 years; 65.7% on DMARDs | Aged ≥ 18 years; Moderate to severe RA for at least 6 months; Recruited through the Harris panel of patients based on pre-registered medical history information. |
| 12) Louder et al., 2016(53) | 380 RA patients; Mean age = 54.9 (SD = 9.3); 81.6% female; Mean disease duration =9.2 (SD= 9.2); | Aged 21 - 80 years; Currently enrolled in a fully insured Humana commercial health plan with medical and pharmacy benefits; at least 2 RA-related medical claims in the previous 12 months, at least 30 days apart, as identified from ICD-9-CM |
| 13) Nolla et al., 2016(33) | 165 RA patients; Mean age = 55.9 (SD = 11.5); 73.8% female; Mean disease duration = 13 (SD =7.8) | Diagnosed with RA for at least 2 years. Currently or previous (<1 year ago) receiving biological agents (BAs) for a minimum of 1 years |
| 14) Ozdemir et al., 2009(34) | 463 RA patients (233 Cheap-talk and 230 Control);Mean age = 53 years (SD = 13); 64% female; Mean disease duration = 8 (SD= 5) | Aged ≥ 18 years; US residents; Self-reported physician diagnosis of RA. |
| 15) Poulos et al., 2014(54) | 836 RA patients; 62.2% = 45-64 years of age; 74.4% female; | Aged ≥ 18 years; Capable of reading and understanding English: living in the US; Self-reported physician diagnosis of RA; Moderate to severe RA symptoms |
| 16) Scalone et al.,2018 (24) | 174 RA patients*; Mean age = 50.0 (SD= 13.6); 57.5% female; Mean disease duration: 8.0 (SD = 8.2); 52.6% had experience with biologic treatment | Aged ≥ 18 years; Formally diagnosed with RA |
| 17) Skjoldborg et al., 2009(35) | 178 RA patients (145 survey 2; 130 survey 3); no participant characteristics reported separately. | Aged 18 - 70; Formally diagnosed with RA and under care of rheumatologist |
| 18) van Heuckelum, et al., 2019(55) | 325 RA patients; Mean age 63.3 years (SD=11.9); 69.2% female; Mean disease duration = 14.7 years (SD=11.2). | Aged ≥ 18 years; Proficiency of the Dutch language; Formally diagnosed with RA by a rheumatologist; Treated with at least one DMARD. |
| 19) Bansback et al., 2016(27) | 2663 General population; Mean age = 44.9 years (SD = 14.8); 56% Female. | Members of a survey panel asked to imagine they have RA |
| 20) Harrison et al.,2015(28) | 733 General population; Mean age = 43.7 years (SD = 15.0); 55% Female | Asked to imagine they have RA |
| **Studies of RA prevention** | | |
| **Source** | **Participant characteristics** | **Inclusion criteria** |
| 21) Finckh et al., 2016(36) | 32 FDRs; Median age 47 years (IQR= 40-60); 79% Female; Mean number of FDRs with RA= 1.2 (SD= 0.5). | Healthy first-degree relatives of patients with RA recruited through an ongoing screening study for preclinical RA. |
| 22) Harrison et al., 2019(38) | 288 FDRs; 66% = 18-39 years of age; 60% Female | Self-reported FDR of someone with RA. |
| 23) Harrison et al., 2020(37) | 30 FDRs+ 78 RA patients; 47%= 40-59 years of age; 86% female | **FDRs**: parents, siblings and adult children of RA patients. No diagnosis of RA  **RA patients:** self-reported physician-confirmed diagnosis of RA. Currently/had been previously treated with a drug to treat RA. |

Disease duration: months or years since diagnosis

*patients characteristics only reported for overall sample of patients which includes RA, PsA, and AS (N total= 508)
